# Polypoidal Choroidal Vasculopathy in Highly Myopic Eyes with Elongated Axial Length

**DOI:** 10.2174/1874364101711010326

**Published:** 2017-11-21

**Authors:** Gregg T. Kokame, Elysse S. Tom, Jessica G. Shantha, Kyle N. Kaneko

**Affiliations:** 1 Division of Ophthalmology, Department of Surgery, University of Hawaii School of Medicine, 651 IIalo St. Honolulu, HI, 96813, USA; 2 The Retina Center at Pali Momi, 98-1079 Moanalua Road, Suite 470, Aiea, HI 96701, USA; 3 Retina Consultants of Hawaii, 98-1079 Moanalua Road, Suite 470, Aiea, HI 96701, USA; 4 Hawaii Macula and Retina Institute, 98-1079 Moanalua Road, Suite 410, Aiea, HI 96701, USA; 5 John A. Burns School of Medicine, University of Hawaii School of Medicine, 651 Ilalo St, Honolulu, HI 96813, USA; 6 Department of Ophthalmology, Emory University, Emory Eye Center, Atlanta, Georgia

**Keywords:** Polypoidal choroidal vasculopathy, High Myopia, Exudative macular degeneration, Photodynamic Therapy, Indocyanine Green Angiography, Bevacizumab, Aflibercept, Ranibizumab

## Abstract

**Purpose:**

To retrospectively review the prevalence of myopia and elongated axial length in eyes with polypoidal choroidal vasculopathy (PCV) and to evaluate treatment response of PCV in highly myopic eyes. PCV has rarely been reported in myopic eyes.

**Methods:**

A retrospective review of all eyes diagnosed with PCV at the clinics of Retina Consultants of Hawaii and the Hawaii Macula and Retina Institute was performed between February of 2007 and April of 2017 to evaluate for eyes with significant myopia and elongated axial length.

**Results:**

A total of 282 eyes were diagnosed with PCV by ICG angiography. There were 144 males (59%) and 99 females (41%). 204 patients had unilateral PCV and 39 patients had bilateral PCV. A total of 3 patients with PCV had significant myopia less than -6 diopters or confirmed elongated axial length. One of these patients had bilateral PCV so there were 4 eyes noted with significant myopia and elongated axial length out of 282 eyes with PCV (1.4%). All 3 patients were Asian and presented with active leakage or bleeding related to PCV diagnosed on indocyanine green angiography and optical coherence tomography. Treatments typically used to treat PCV including intravitreal antiangiogenic medications and photodynamic therapy were utilized.

**Conclusion and Importance:**

High myopia is rare in eyes diagnosed with PCV, even though choroidal neovascularization is a common cause of vision loss in myopic macular degeneration. However, even in highly myopic eyes, PCV may show signs of resistance to antiangiogenic medications.

## 1 INTRODUCTION

Polypoidal Choroidal Vasculopathy (PCV) is a variant of neovascular age related macular degeneration. The subretinal neovascularization is type I according to the classification system of Gass [1]. Type I subretinal neovascularization, including PCV, is due to abnormal blood vessels growing below the retinal pigment epithelium and above Bruch’s membrane [2]. The subretinal neovascularization is made up of polypoidal vascular dilations with or without a Branching Vascular Network (BVN) [2].

Choroidal Neovascularization (CNV) in highly myopic eyes is not uncommon, but is usually related to myopic macular degeneration [3]. In fact PCV has been shown to be an extremely rare cause of choroidal neovascularization in myopic eyes. In a large study of CNV in myopic eyes looking specifically with indocyanine green angiography (ICG) for PCV, Kang and Koh did not find even one case of PCV in a series of 297 eyes with treatment-naïve myopic choroidal neovascularization [4]. Although high myopia is a significant cause of visual impairment worldwide, it lacks a standard definition [5]. Common previous markers have included refractive error < -6.0 D or -8.0 D, or elongated axial length [5]. Elongated axial length is an endophenotype of myopia and the primary determinant of myopia [6]. In a recent study of highly myopic eyes defined as -6 D or greater, axial length was noted to be on average 26.56 mm and to range from 24.95 mm to 38.86 mm as compared to an average of 23.71 mm in normal controls [7]. In this retrospective study of PCV, herein we report on the clinical characteristics of PCV noted on both Optical Coherence Tomography (OCT) and Indocyanine Green Angiography (ICGA) in highly myopic eyes based on a refractive error of -6 diopters or greater with corresponding elongated axial length.

## 2 METHODS

This study involved a retrospective review of all patients seen with a diagnosis of Polypoidal Choroidal Vasculopathy (PCV) diagnosed by ICG angiography between February of 2007 and April of 2017 at the clinics of Retina Consultants of Hawaii and the Hawaii Macula and Retina Institute. Since PCV has been rarely reported in eyes with significant myopia [3], and because high myopia lacks a standard universally agreed upon definition, eyes were included in this study if myopia was < -6.0 diopters in phakic eyes or had a confirmed elongated axial length of 24.95 mm or more based on the study by Wang and colleagues [7], which correlated to findings in eyes with < -6.0 D. All eyes included in this high myopic category had typical funduscopic features of high myopia including peripapillary atrophy or myopic crescent. This retrospective study was deemed exempt from Institutional Review Board (IRB) approval by the Western Institutional Review Board (#1-1019457-1), and the study was conducted in accordance with the declarations of Helsinki.

Patient data was evaluated to include the age and sex of patients, the refractive error, the funduscopic findings, the optical coherence tomography findings, the Fluorescein Angiogram (FA) findings and the indocyanine green angiography used to confirm the diagnosis of PCV. Axial length data were obtained retrospectively in all myopic eyes to confirm an elongated axial length usually associated with eyes with high myopia of < -6 diopters or more.

## 3 RESULTS AND PATIENTS

Between February of 2007 and April of 2017 at the clinics of Retina Consultants of Hawaii and the Hawaii Macula and Retina Institute there were 282 eyes diagnosed with PCV by ICGA on retrospective review of clinic records. 204 patients had unilateral PCV, and 39 patients had bilateral PCV, so in total there were 282 eyes of 243 patients with a diagnosis of PCV. The average age was 77.6 years with a range in age from 47 to 102 years. There were 144 males (59%) and 99 females (41%). Elongated axial length and high myopia was documented in 4 of the 282 eyes (1.4%) with a diagnosis of PCV by ICGA. Two of the myopic patients had unilateral PCV and one myopic patient had bilateral PCV. Herein are the three patients described with PCV in highly myopic eyes.

### 3.1 Patient 1

**Fig. (1):**
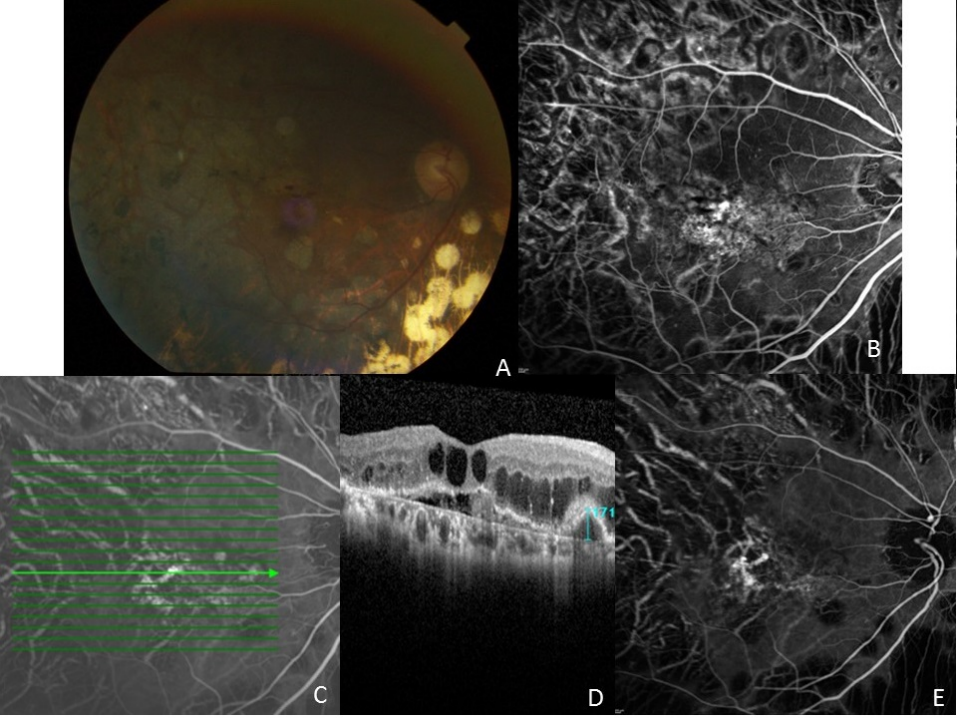
(A) Fundus photograph of the right eye of patient 1. Note the small subretinal hemorrhage within the macula and peripheral panretinal laser photocoagulation for proliferative diabetic retinopathy. (B) Fluorescein angiography shows leakage in the inferotemporal fovea. (C, D) ICG angiogram showing PCV complex with nasal polyp and horizontal line showing ICG location for optical coherence tomography line scan. Note the characteristic inverted U-shaped polyp with a height of 171 um, the adjacent branching vascular network (shallow elevation of the RPE), and diffuse intraretinal edema with serous detachment. (E) ICG angiography shows multiple hyperfluorescent polyps with associated branching vascular network.

A 73-year-old Chinese female presented with vision loss in her right eye with thin subretinal hemorrhage. The anterior segment exam of the right eye showed a well centered posterior chamber implant. Funduscopic examination showed a thin subretinal hemorrhage in the macula with superior edema with cystic changes (Fig. **1A**). Fluorescein angiography showed a blocking defect from the blood and occult subretinal leakage centrally without any evidence of retinal vascular leakage (Figs. **1B**-**D**). Her axial length was 25.21 mm by ultrasound biometry confirming an elongated myopic eye. Her past medical history included non-insulin dependent diabetes mellitus and quiescent proliferative diabetic retinopathy with previous macular laser and panretinal laser photocoagulation (PRP). Her vision in the right eye was as good as 20/25 prior to a vitreous hemorrhage, which required vitrectomy, removal of epiretinal membranes and endolaser PRP in 2005. She presented with subretinal hemorrhage in the right eye five years following the vitrectomy. ICG angiography showed a central hyperfluorescent branching vascular network with multiple hyperfluorescent polyps classic for PCV (Fig. **1E**).

On OCT there were multiple inverted U-shaped polyps with diffuse intraretinal edema (Fig. **1D**). Her subfoveal choroidal thickness was 170 um, which is normal and not thick as often seen in PCV. Initial treatment for the subretinal neovascularization was performed with four intravitreal bevacizumab injections and two ranibizumab injections with a poor response with persistent macular edema. Two photodynamic therapy treatments combined with injections of intravitreal bevacizumab 1.25 mg and dexamethasone 400 ug were performed with resolution of leakage and macular edema with a final visual acuity of 1/200. Polypoidal choroidal vasculopathy was not present in the left eye on ICG angiography.

### 3.2 Patient 2

**Fig. (2):**
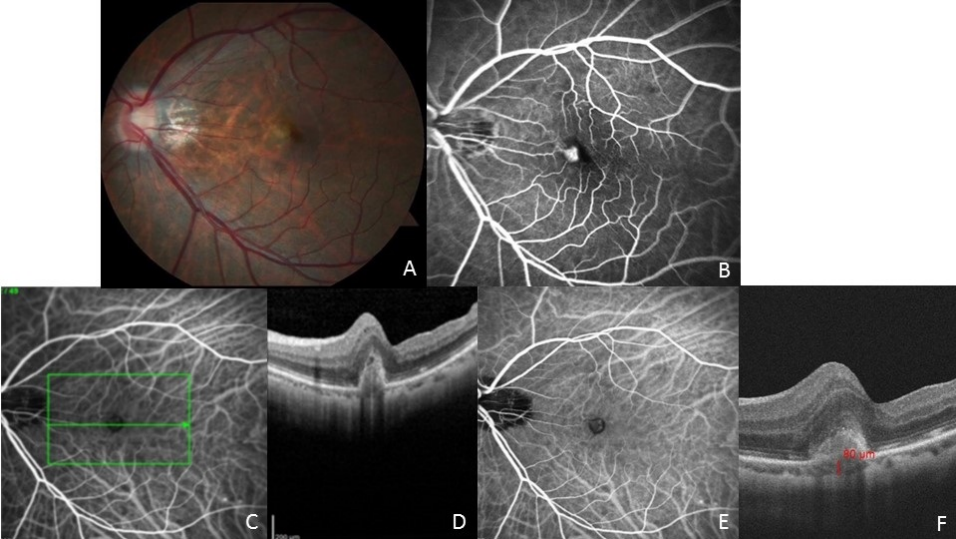
(A) Fundus photograph of the left eye of patient 2 shows nasal retinal thickening. (B) Fluorescein angiography shows leakage in the area of nasal retinal thickening (C, D) ICG angiogram showing central hyperfluorescent lesion with hypofluorescent border and a horizontal line showing ICG location for optical coherence tomography line scan. Note the characteristic inverted U-shaped polyp overlying macular edema and cystic change. (E) Indocyanine green angiography shows a hyperfluorescent polyp with a surrounding hypofluorescent halo. (F) Choroidal thickness measurement on OCT confirming thin choroid (80 microns) underneath the fovea.

A 66-year-old Japanese female presented with blurred vision in the left eye for six months and a visual acuity of 20/80. Her past ocular history in the left eye included prior vitrectomy with membrane peeling, and cataract extraction. Anterior segment examination was significant for a well-centered posterior chamber IOL with central capsulotomy. Funduscopic exam showed nasal RPE thickening and edema without subretinal hemorrhage (Fig. **2A**). FA showed nasal focal leakage in the area of edema (Fig. **2B**). ICG angiography showed an inferonasal hyperfluorescent polyp with a surrounding hypofluorescent ring (Figs. **2C**, **E**). Optical coherence tomography showed macular cystic changes overlying a nasal inverted U-shaped elevation of the retinal pigment epithelium typical for a polyp (Fig. **2D**). The choroidal thickness was thin typical of highly myopic eyes (subfoveal choroidal thickness of 80um) (Fig. **2F**), and similar to the fellow eye. Her axial length was 27.95 mm by ultrasound biometry consistent with an elongated highly myopic eye. She received seven intravitreal bevacizumab injections with improvement in vision to 20/25 and resolution of edema, which was then maintained without treatment for eight months. There was no PCV in the right eye on ICG angiography.

### 3.3 Patient 3

**Fig. (3):**
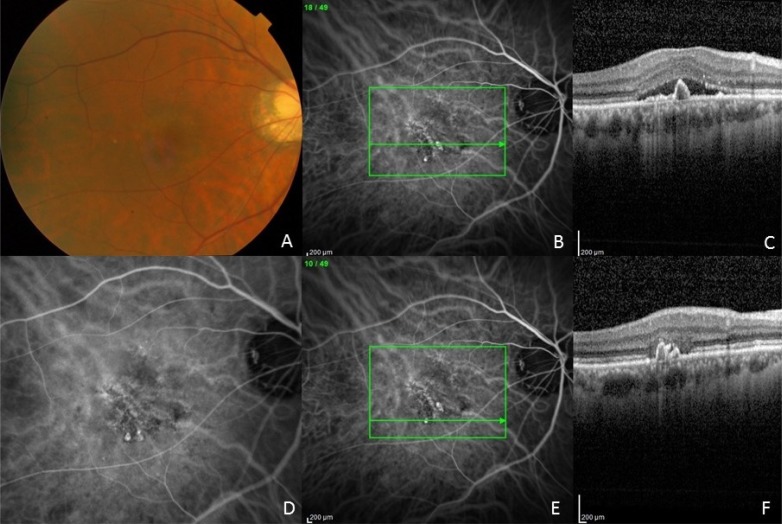
(A) Fundus photograph of the right eye of patient 3. Note the myopic fundus with peripapillary RPE atrophy and laquer cracks and serous macular detachment. (B, C) ICG angiography showing PCV complex with polypoidal dilations and branching vascular network. Note the inverted U-shaped polyp and adjacent branching vascular network with shallow elevation of the RPE. There is overlying serous detachment. (D) ICG angiogram showing magnified view of PCV complex with branching vascular network and terminal polyps. (E, F) ICG angiogram of PCV complex with horizontal line scan through polyps inferiorly outside the area of leakage. Note the multiple inverted U-shaped polyps and loss of the photoreceptor layer above the polyps.

A 75-year old Chinese male presented with blurred vision in the right eye for 6 months and a visual acuity of 20/50. Visual acuity in the left eye was 20/20. Refraction was OD: -9.50+0.75 x 005 OS: -9.75+0.75 x 173. Anterior segment examination was significant for a moderate nuclear sclerotic cataract in both eyes. Funduscopic examination showed a myopic fundus with peripapillary RPE atrophy and laquer cracks in both eyes. There was subretinal fluid in the right eye (Fig. **3A**). Fluorescein angiography showed occult leakage temporally in the right eye, no leakage in the left eye, and hyperfluorescent laquer cracks in both eyes. ICG angiography showed inferotemporal polyps with a branching vascular network in the right eye (Figs. **3B**, **D**, **E**) and a PCV complex in the left eye. OCT showed subretinal fluid centrally (Fig. **3C**) and typical inverted U-shaped polyps (Figs. **3C**, **F**). The choroidal thickness on OCT was 310 um in the right eye and 282 um in the left eye. The axial length was 28.95 mm in the right eye and 28.38 mm in the left eye by ultrasound biometry. He initially received 4 sequential intravitreal bevacizumab injections in the right eye, but had persistent subretinal fluid, and was treated with intravitreal aflibercept. Subretinal fluid decreased but persisted, and a repeat ICG angiogram showed persistent polyps and branching vascular network. After 28 treatments with intravitreal aflibercept there was persistent shallow subretinal fluid, but visual acuity remained 20/50. The PCV complex in the fellow left eye was not actively leaking and did not require treatment.

## 4 DISCUSSION

Polypoidal choroidal vasculopathy is rare in eyes with high myopia, but in this retrospective study there were definite cases of PCV in eyes with elongated axial length and high myopia making up 1.4% of the cases diagnosed with PCV by ICGA over a 10-year period. These myopic eyes with PCV received the usual treatments provided for exudative macular degeneration, including initial treatment with antiangiogenic drugs. For patient 2 the leakage responded well to intravitreal bevacizumab, and the treatments were eventually discontinued without recurrence. This patient also had a very thin choroid with a subfoveal choroidal thickness of just 80 um (Fig. **2F**). High myopic eyes tend to have thinner choroid, which increases with elongated axial length [7, 8]. The normal mean subfoveal choroidal thickness is 272 um with a range of 191 um to 353 um [9], so the choroidal thickness in patient 2 was significantly thin. As can be seen with exudative myopic macular degeneration, which is often associated with a thin choroid, antiangiogenic medications can sometimes be tapered off without recurrence of leakage or bleeding.

PCV has a higher risk of resistance to antiangiogenic drugs [10, 11], but it is uncertain whether or not this resistance is a potential problem in PCV in highly myopic eyes, which have a thinner choroid and an elongated axial length. In patient 1 there was a poor anatomic response to treatments with intravitreal bevacizumab and ranibizumab, so photodynamic therapy was required to resolve the macular edema and active leakage. For Patient 3 there was persistent subretinal fluid even after 4 injections of intravitreal bevacizumab and 28 injections of intravitreal aflibercept at frequent and consistent intervals, indicating resistance to antiangiogenic medications with a poor anatomic response, although vision has been maintained. These cases demonstrate that there may be an increased risk of resistance to antiangiogenic drugs in PCV even in eyes with high myopia and elongated axial length. Because of the decreased response to therapy noted in eyes with PCV, diagnostic testing to rule out the diagnosis of PCV, including the gold standard of ICG angiography [12], should be considered in exudative macular degeneration in myopic eyes, especially when there is persistent disease activity after treatment.

Axial length is the main determinant of refractive error and high myopia. Elongated axial length is an endophenotype of myopia, and all eyes in this series had elongated axial lengths ranging from 25.21 to 28.95 mm (average= 27.6 mm), which are above the lower range (24.95 mm) noted in eyes with high myopia of less than -6 diopters [7]. The polypoidal choroidal vasculopathy was typical with the subretinal neovascularization occurring between the retinal pigment epithelium and Bruch’s membrane on OCT [2] and visible as hyperfluorescent polypoidal dilations with a hypofluorescent halo on ICG angiography. In a retrospective cross-sectional study in South Korea, evaluating myopic CNV in 297 eyes with ICG angiography, none of the patients had polypoidal lesions [4]. These studies support that PCV does not develop commonly in highly myopic eyes. Even though PCV is more common in Asian populations [13], and all of the PCV cases in these myopic eyes were in East Asian patients, and even though high myopia is more prevalent in Asia [14], PCV is only rarely seen in highly myopic eyes. Most eyes with PCV have a thick choroid [15]. In Patients 1 and 2, as well as in other highly myopic eyes, the choroid is very thin [7, 8], although patient 3 had choroidal thickness well within the normal range. We speculate that the thin choroid in most myopic eyes could be a factor in the low prevalence of PCV in highly myopic eyes.

In a previous multicenter study by Mauget-Faysse *et al.* [16] PCV was noted to be associated with the tilted disc syndrome or high myopia with a posterior staphyloma in 8 eyes. In 5 of the 8 eyes PCV was documented on ICG angiography in highly myopic eyes, but none of these cases had a documented elongated axial length. The authors previously postulated that PCV developed at the border of hypoplastic and normal choroid at the edge of a posterior staphyloma, but this was not noted in our cases, which had high myopia with an elongated axial length without a posterior staphyloma. To the best of our knowledge our cases are the first reports of PCV in highly myopic eyes without a posterior staphyloma. Although Mauget-Faysse thought PCV was a complication associated with tilted disc syndrome and posterior staphyloma (16), we feel that the development of PCV is rare in myopic eyes, and that the thin choroid could potentially be a protective factor against the development of PCV.

Treatment in Patient 2 was performed with antiangiogenic therapy alone and was successful at resolving the edema and improving vision to 20/25 from 20/80 without laser or photodynamic therapy. In the series by Mauget-Faysse treatment was only performed with laser or photodynamic therapy in their cases [16]. This successful treatment reported herein with antiangiogenic therapy in patient 2 does indicate that antiangiogenic therapy for PCV in myopic eyes can be successful, and since myopic eyes can have spontaneous resolution [3], it is reasonable to follow these eyes after regression of the active lesions without treatment on an as needed basis for treatment. A thin choroid as noted in patient 2 may be a possible positive predictive factor for following patients as needed after a good anatomic response to antiangiogenic drugs. However, patients 1 and 3 both showed a poor response to antiangiogenic therapy, and highlight the potential need to consider other treatment options including photodynamic therapy or combination photodynamic therapy with antiangiogenic therapy with or without steroids. The use of combination therapy with photodynamic therapy and antiangiogenic therapy for myopic choroidal neovascularization is supported by Rishi and colleagues, which showed better visual outcomes with combination therapy than with photodynamic monotherapy or antiangiogenic monotherapy [17].

## CONCLUSION

PCV is rare in highly myopic eyes occurring in 1.4% of eyes diagnosed with PCV. However, PCV with typical features on ICG angiography of polypoidal dilations and a branching vascular network (Figs. **3B**, **D**, **E**), as well as typical OCT findings of the double line sign showing the branching vascular network and the inverted U-shaped elevations of the retinal pigment epithelium typical of polyps (Figs. **3C**, **F**) can occur in highly myopic eyes with elongated axial length. PCV lesions in highly myopic eyes can show resistance to antiangiogenic therapy, as noted in PCV lesions in general, which may require alternative treatment options. Appropriate diagnostic testing including ICG angiography to make the diagnosis of PCV is important, especially when there has been a poor response to antiangiogenic therapy.
